# Deficient copper concentrations in dried-defatted hepatic tissue from *ob/ob* mice: A potential model for study of defective copper regulation in metabolic liver disease

**DOI:** 10.1016/j.bbrc.2015.03.067

**Published:** 2015-05-08

**Authors:** Stephanie J. Church, Paul Begley, Nina Kureishy, Selina McHarg, Paul N. Bishop, David A. Bechtold, Richard D. Unwin, Garth J.S. Cooper

**Affiliations:** a Centre for Advanced Discovery and Experimental Therapeutics, Central Manchester University Hospitals NHS Foundation Trust (CMFT), Manchester M13 9WL, United Kingdom; b Centre for Endocrinology & Diabetes, Institute of Human Development, Manchester Academic Health Science Centre, University of Manchester, Manchester, United Kingdom; c School of Biological Sciences, Faculty of Science, and Department of Medicine, Faculty of Medical and Health Sciences, University of Auckland, Auckland, New Zealand; d Faculty of Life Sciences, University of Manchester, Manchester M13 9PT, United Kingdom; e Department of Pharmacology, Medical Sciences Division, University of Oxford, Oxford OX1 3QT, United Kingdom

**Keywords:** Obesity, Diabetes, Liver, Copper, Non-alcoholic fatty liver disease, Non-alcoholic steatohepatitis

## Abstract

*Ob/ob* mice provide an animal model for non-alcoholic fatty liver disease/non-alcoholic steatohepatitis (NAFLD/NASH) in patients with obesity and type-2 diabetes. Low liver copper has been linked to hepatic lipid build-up (steatosis) in animals with systemic copper deficiency caused by low-copper diets. However, hepatic copper status in patients with NAFLD or NASH is uncertain, and a validated animal model useful for the study of hepatic copper regulation in common forms of metabolic liver disease is lacking. Here, we report parallel measurements of essential metal levels in whole-liver tissue and defatted-dried liver tissue from *ob/ob* and non-obese control mice. Measurements in whole-liver tissue from *ob/ob* mice at an age when they have developed NAFLD/NASH, provide compelling evidence for factitious lowering of copper and all other essential metals by steatosis, and so cannot be used to study hepatic metal regulation in this model. By marked contrast, metal measurements in defatted-dried liver samples reveal that most essential metals were actually normal and indicate specific lowering of copper in *ob/ob* mice, consistent with hepatic copper deficiency. Thus *ob/ob* mice can provide a model useful for the study of copper regulation in NAFLD and NASH, provided levels are measured in defatted-dried liver tissue.

## Highlights

• *Ob/ob* mice develop hepatic copper deficiency.
• Copper deficiency develops alongside NAFLD/NASH.
• Metal measurements in whole tissue are impacted by infiltrating fat.
• Levels of all essential metals are lower in *ob/ob* mice when measured in whole liver.
• After fat extraction, metal levels are normal, with the exception of copper.

## 1 Introduction

Mice homozygous for the spontaneous mutation obese (Lep^ob^, *ob/ob*), which leads to absolute deficiency of the adipocyte-derived hormone leptin, exhibit hyperphagia and obesity accompanied by a type-2 diabetes-like syndrome comprising glucose intolerance and hyperglycaemia [1,2]. In these mice, the abnormal metabolic state is accompanied by several endocrine defects, including elevated levels of the pancreatic beta-cell hormones insulin and amylin [3], insulin resistance, and deficiency of the second major adipocyte-derived hormone, adiponectin [2,4]. Obesity in *ob/ob* mice is accompanied by an increase in both the number and size of adipocytes; however, although hyperphagia contributes to this obesity, homozygous animals gain excess weight and deposit excess fat when restricted to a diet sufficient for normal weight maintenance in matched non-affected controls [1].

Non-alcoholic fatty liver disease (NAFLD) is a common form of fatty liver disease that is not caused by excess alcohol intake and is associated with obesity and insulin resistance. NAFLD can progress to Non-alcoholic steatohepatitis (NASH), where the steatosis is associated with inflammation and fibrosis, and this in turn can progress to liver cirrhosis. The *ob/ob* mouse develops hepatic steatosis that worsens with age, from a syndrome resembling NAFLD in humans to one mirroring NASH [4]. Adiponectin deficiency makes a substantive contribution to the development of NAFLD and NASH in *ob/ob* mice, and hepatic structure and function are largely restored by adiponectin treatment [4]. However, adiponectin replacement is not a therapeutic option at present, owing to the great structural complexity of the hormone and the resulting inability to produce pure hormone for therapeutic application [5–7].

Over the last decade, our group has identified and characterised a pathogenic disorder of copper regulation, which occurs in diabetic patients [8,9] and animals [10–13], and which causes systemic copper overload accompanied by copper imbalance: this manifests as copper overload in the kidneys [14] but localized copper deficiency in other organs, for example as left-ventricular copper deficiency in diabetic cardiomyopathy [13]. Reversal of this systemic copper imbalance in patients or animals can be accomplished by treatment with the Cu(II)-selective chelator, triethylenetetramine (TETA), which restores systemic copper balance [8] and normalizes organ copper content and function, for example in the myocardium [9,13] and kidneys [14]. Hepatic steatosis also occurs in animals with low dietary copper intakes [15] but the hepatic copper status in patients with NAFLD or NASH remains to be accurately determined. Importantly, patients and animals with NAFLD or NASH do not develop typical signs of systemic copper deficiency, which include low plasma copper, low urinary copper output, low concentration and activity of plasma caeruloplasmin, anaemia, neutropenia, and low erythrocyte superoxide dismutase activity [16].

A recent report has described lowered hepatic copper concentrations in patients with NAFLD and NASH [17]. The authors interpreted their data to show that lower hepatic copper levels in patients were associated with more pronounced hepatic steatosis, NASH, and components of the metabolic syndrome [17]. Furthermore, they also reported development of hepatic steatosis and insulin resistance in response to dietary copper restriction in rats, which was interpreted to suggest that impaired copper availability may be involved in the development of NAFLD. Accordingly, they hypothesized that low hepatic copper might contribute to the disorder of hepatic lipid metabolism in clinical NAFLD and NASH. However, organ infiltration with elevated concentrations of lipid causes an excluded-volume effect, for example with respect to metals ions, that leads to spuriously low measured values for tissue metals because they are excluded from the expanded lipid compartment in which they are insoluble; this process is exemplified by factitious hyponatraemia caused by hypertriglyceridaemia [18]. In these abovementioned studies of copper in NAFLD/NASH, hepatic copper levels were determined in non-defatted, dried hepatic tissue, as is done for the diagnosis of Wilson's disease [17,19]. Drying alone does not, however, remove neutral lipid from tissues. Furthermore, Wilson's disease leads to marked hepatic copper overload with hepatitis, but does not lead to significant steatosis [19], and must therefore be differentiated from NASH, which does [4]. A method suitable for the measurement of elevated copper levels in Wilson's disease is thus unlikely to be applicable for the accurate measurement of deficient copper levels in NAFLD/NASH, where steatosis is a probable cause for spurious lowering of metal levels.

It is uncertain whether elevated hepatic lipid levels are sufficient to cause factitious lowering of metal levels in *ob/ob* mice, or whether these mice develop hepatic copper deficiency. Therefore, we measured metal levels in untreated liver tissue from *ob/ob* and control mice, and compared them with levels from the corresponding tissue samples after they had first been dried and defatted. Reported measurements of hepatic copper in patients with NAFLD or NASH have to date been performed only on dried hepatic tissue that had not been defatted [17], so it remains to be determined whether the reported low hepatic copper levels reflect the actual hepatic copper status in such patients, or rather whether any apparent lowering of hepatic copper is merely factitious.

As a necessary pre-translational stage in the process of proving that low copper levels might cause damage to a particular organ, it is necessary to develop an informative animal model. Here, we describe studies in which we have measured hepatic copper levels in the livers of *ob/ob* mice at 11 weeks of age, when they have developed marked NAFLD/NASH as shown by numerous histological and biochemical indices of disease, as we have previously shown [2,4]. We quantitated metals in whole hepatic tissue (mol/kg wet-weight) and in defatted-dried tissues (mol/kg defatted-dried weight) from the same individuals: the former measurements aimed to determine whether metals in mice with hepatic steatosis are subject to factitious lowering, and the latter to ascertain whether any such effect can be negated by drying/defatting, and consequently whether any actual abnormalities in the regulation of any essential metals could be identified.

We found evidence for a marked excluded-volume effect in untreated hepatic tissue from *ob/ob* livers that was resolved in defatted-dried tissue. Thus, it proved necessary to measure copper levels in defatted-dried tissue rather than in untreated-liver tissue, in which major amounts of neutral lipid evidently cause spurious lowering of metal content due to an excluded-volume effect. Hepatic Cu levels were markedly lower in defatted-dried tissue from *ob/ob* mice, 339 (316-361) μmol/kg defatted-dried wt (mean ± 95% confidence interval, CI) as compared to values in non-obese controls, 502 (406-597); *P* = 0.0053. These data also provide strong evidence for specificity in that copper was the only trace metal to show substantive lowering in the livers of *ob/ob* mice.

We conclude: (1) that *ob/ob* mice develop an actual (that is, non-factitious) and specific (with respect to other physiological metals) hepatic copper deficiency state wherein levels are decreased to ∼65% of normal; and (2) that these mice can serve as a suitable model for more in-depth nonclinical studies of copper dysregulation in NAFLD and NASH to determine whether this degree of lowering of tissue copper might impair hepatic lipid regulation. Further studies are required as a prelude to any possible future clinical interventional studies.

## 2 Materials and methods

### 2.1 Ethics

All animal procedures were licensed by the UK Home Office, approved by the Institutional Animal Ethics Committee, and conducted in accordance with the Animals (Scientific Procedures) Act (1986). Study performance was consistent with the Guide for the Care and Use of Laboratory Animals [20], and this report is consistent with the ARRIVE guidelines for the reporting of animal research [21].

### 2.2 Animal model

Male *ob/ob* mice in the C57BL/6 background (N = 10) and C57BL/6 background controls (N = 7) were from Harlan Laboratories (Hillcrest, UK) and were studied at 11–12 weeks of age. We have previously shown that *ob/ob* mice at this age demonstrate marked hepatic fat infiltration with clear signs of NAFLD/NASH [2,4]. Animals were maintained under a 12:12 h light:dark cycle and housed at 22 ± 2 °C and 60% humidity, with *ad libitum* access to standard chow (Maintenance Rodent Diet; Special Diet Services, Essex, UK) and fresh water. This diet provides copper intakes consistent with known physiological requirements [22], so animals were not subject to inadequate dietary copper. On the final experimental day, animals were killed by cervical dislocation followed by decapitation, and livers excised and stored at −80 °C until analysis.

### 2.3 Preparation of liver extracts

Metal concentrations were determined as described here, either on a wet-weight basis from unmodified tissue or from tissue that had first been dried and defatted.

For determination on a fresh-weight basis, samples of liver tissue (50 ± 5 mg wet-weight) which had first been blotted dry, were digested as described below. For dried-defatted tissue measurements, aliquots of 150 mg wet weight (±5%) were dried to constant weight in a centrifugal concentrator (Savant Speedvac; Thermo-Fisher, Waltham, MA).

Lipids were extracted in 2 ml 50:50 (v/v) chloroform (Acros Organics 32667 “ECD tested for pesticide analysis”, Acros Organics, Geel, Belgium):2,2,4-trimethylpentane (Fluka 34499 “Pestanal” Grade, Sigma Aldrich, Gillingham, UK) using a TissueLyser II (Qiagen, Manchester, UK). A 3-mm Tungsten carbide bead was added to each sample tube and samples were extracted for 10 min at 25 Hz. Samples were vortex-mixed for 10–15 s prior to centrifugation at 2400 g for 15 min, and supernatants were removed from the remaining tissue. Samples were then washed with 1 ml methanol (Fluka 34966 Chromasolv LC-MS Grade, Sigma) and centrifuged at 2400 g for 15 min, supernatants removed, and samples dried overnight. Aliquots of 10 mg (dried-delipidated weight) were transferred into 2-ml microcentrifuge tubes (Eppendorf) for acid digestion.

### 2.4 Digestion

Reactions were performed in Trace Metal Grade concentrated nitric acid (A509 Trace Metal Grade; Fisher, Loughborough, UK) to which had been added (5% v/v) Agilent Internal Standard mixture (5183-4681; Agilent Technologies, Cheadle, UK). This internally-standardised acid was also used at appropriate dilutions to provide rinse and calibration solutions, at 2% (v/v) final nitric acid. Calibration solutions were produced by appropriate dilutions of Environmental Calibration Standard (Agilent 5183-4688).

Acid digestion was carried out using a simple ‘open vessel’ method. Tissue aliquots were briefly centrifuged to ensure that the tissue sat at the bottom of the tube. The tube lids were punctured to prevent pressure build up, and 0.2 ml standard-containing nitric acid added. Tubes were then inserted into a “Dri-block” heater which was initially at room temperature. Tubes containing standard-containing acid but no sample were processed in each batch to provide “digestion” blanks. Temperature was then set to 60 ^°^C and the block switched on. After 30 min, the set temperature was increased to 100 ^°^C, and digestion continued for a further 210 min. After digestion, the tubes were allowed to cool overnight. 100 μl aliquots of the digestion solution were then added to 15 ml Falcon tubes (Greiner) containing 5 ml LC-MS grade water, to produce solutions for analysis at a final nitric acid concentration of 2% (v/v).

### 2.5 Metal measurements

Tissue metal concentrations were measured using an Agilent 7700x ICP-MS spectrometer with a multi-element method including all elements present in the calibration solution. A multi-element method was written including all components present in the calibration solution, and following Agilent's recommendations for operation modes, integration times and internal standard assignment.

For each analytical batch, multi-element calibration was performed using serial dilutions of the calibration standard. An intermediate concentration from this calibration series was used as a periodic quality control (QC) sample throughout each analytical batch. Instrument and digestion blanks were also interspersed through each set of randomised samples. Signals derived from non-essential elements were identified by comparison of blank, calibration and sample concentrations, and eliminated prior to reporting. We verified that the copper content of the animals’ drinking water was on average ∼5.2 μg/l (i.e. >100-fold below the maximum of 2 mg/l currently allowed in potable drinking water for humans by current UK guidelines; http://www.dwi.gov.uk).

### 2.6 Statistical methods

Tabulated data are means (±95% CI). Significance of between-group contrasts was determined by Welch's modified *t*-test to allow for unequal variances. Statistical calculations were performed using S-plus v8.1 (Spotfire; TIBCO, Somerville, MA), and graphs were plotted using GraphPad v6.04 (Prism; La Jolla, CA). *P*-values of <0.05 have been considered significant, and those of 0.05 ≤ *P* < 0.10 have also been tabulated.

## 3 Results and discussion

We first measured the concentrations of 10 essential metals in unmodified liver tissue from 10 *ob/ob* mice and seven controls by using ICP-MS with reference isotopes for each element as shown (Table 1). Metal concentrations were uniformly greater in control than obese mice. The mean (±range) of ratios of metal concentrations between control and obese mice was 1.4 (1.1-1.9): therefore, ratios of concentrations between control and *ob/ob* mice were ∼similar for all essential metals measured.

We next repeated metal measurements in liver tissue from the same animals after samples had been dried to constant weight and defatted (Table 2). Under these conditions, tissue copper concentrations were significantly greater in control mice, 502 (406-597) μmol/kg dried-defatted tissue, than in *ob/ob* mice, 339 (316-361); the average ratio in copper concentrations between control and *ob/ob* mice was 1.5 (*P* = 0.0053). By contrast, ratios in levels of other essential metals did not differ significantly between the groups, except in the case of sodium where the difference was of borderline significance (*P* = 0.047), and manganese, which also showed a minor yet significant decrease.

**Fig. 1:**
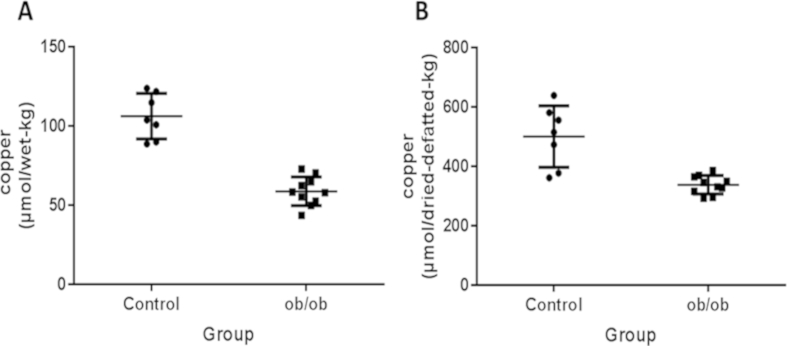
Copper concentrations in hepatic tissue from *ob/ob* (N = 10) and C57BL/6 contol (N = 6) mice. (A) Copper concentrations in unmodified hepatic tissue. (B) Copper concentrations in hepatic tissue that had been dried to constant weight and defatted prior to analysis. Copper was measured by inductively-coupled plasma mass spectrometry. Long-thin horizontal lines indicate mean values, and short-thick lines ± standard deviation.

Plots of copper concentrations in whole-liver tissue and in dried-defatted liver tissue are shown in Fig. 1A, B. Copper concentrations were increased in dried-defatted tissue by an average of 4.7-fold in controls and 5.7-fold in *ob/ob* mice (Fig. 1B) compared with corresponding values in whole-liver tissue (Fig. 1A). Copper concentrations in dried-defatted tissue were markedly lower in *ob/ob* mice as compared with matched controls (Fig. 1B), providing substantive evidence for genuine hepatic copper deficiency. By contrast, the data in Fig. 1A were confounded by an excluded-volume effect, as shown by comparison of the data in Tables 1 and 2

Thus *ob/ob* mice demonstrated hepatic copper levels that were significantly lower than those in controls on the basis of measurements in dried-defatted tissue, which are informative. This difference was present at a time when these mice are known to have hepatic steatosis and inflammation (NAFLD/NASH) [2,4]. Comparison between the pattern for copper and those for other metals indicates that this deficiency is likely to occur through a process specific for copper, as opposed to any of the other essential metals. The exact molecular mechanism by which copper becomes deficient in the livers of *ob/ob* mice is uncertain at present, but could mirror that which causes myocardial copper deficiency and cardiac damage in diabetic cardiomyopathy, where there is substantive evidence that diabetes can evoke regulatory defects in many of the pathways that mediate myocellular copper uptake and distribution [13].

Here, hepatic copper deficiency in *ob/ob* mice was unmasked by measuring metal levels in defatted-dried tissue (Table 2): this obviated the interference that was caused by the excluded-volume effect of elevated hepatocellular lipids (HCL) when metal levels were measured in unmodified hepatic tissue (Table 1). These findings show that subsequent studies of dysregulated copper metabolism in the livers of *ob/ob* mice, and related obese or diabetic states in animals and patients with hepatic steatosis, will need to be undertaken using dry-defatted liver tissue.

Copper deficiency can cause neurodegeneration as well as cardiovascular and haematological disorders (reviewed in Ref. [23]) via impairment of cellular antioxidant defences and concomitant defects in copper-mediated processes and pathways [13]: these effects are thought to cause formation of excessive amounts of reactive oxygen species (ROS) in affected tissues. Identified pathogenic mechanisms include defective superoxide clearance caused by impaired copper metalation and activity of superoxide dismutase 1 (SOD1) [13], a copper/zinc enzyme; and defective activity of cytochrome c oxidase subunits CO1 and CO2 - both of which are copper enzymes (reviewed in Ref. [24]).

Hepatic steatosis is defined by an increased content of HCL and is frequently observed in the context of insulin resistance, for example in obesity and type-2 diabetes. Elevations in HCL are generally thought to result from dietary excess of saturated fat uptake coupled with defective hepatic lipid utilisation and clearance [25]. However, hepatic steatosis is also well known to occur as a result of toxic injury to the liver, for example that caused by exposure to halogenated hydrocarbons such as carbon tetrachloride: such processes can also cause marked elevations in HCL through the formation of free radicals that damage hepatocytes, thus preventing them from synthesising lipoprotein from triglycerides [26]. Therefore, it is possible that copper deficiency could contribute to or cause liver injury via enhanced tissue ROS formation rather than simple ‘spill-over’ of excess dietary lipid.

Our data are consistent with the idea that the lowered levels of hepatic copper measured here in *ob/ob* mice could be sufficient to contribute to the hepatic steatosis and inflammation that occur in these mice. Hepatic steatosis has been linked to low liver copper levels in animals receiving low-copper diets [27,28]. Thus, it is possible that hepatic copper deficiency could be a contributory cause rather than a consequence of hepatic steatosis in obesity and diabetes: this state may not be caused solely by elevated dietary lipid intake or defective hepatic lipid clearance. Rather, it may represent a manifestation of liver injury caused by hepatic copper deficiency leading to organ pathology via mechanisms similar to those by which diabetes causes myocardial copper deficiency and cardiomyopathy [13].

In summary, we report here that *ob/ob* mice develop substantively deficient hepatic copper levels at an age at which they manifest signs of metabolic liver disease. This localised copper-deficiency state appears to be specific for copper as opposed to other essential metals and occurs in animals that do not display signs of systemic copper homeostasis. Therefore, the hepatic copper deficiency in *ob/ob* mice reflects a localised process. The molecular mechanisms responsible for hepatic copper deficiency in *ob/ob* mice could reflect those that cause myocardial copper deficiency and tissue damage in diabetic cardiomyopathy. Whether low hepatic copper levels occur in patients with NAFLD or NASH, and could contribute to the pathogenesis of these diseases in animals or patients, remains to be determined.

## Author information

GJSC is named as inventor on patents disclosing Cu-selective chelators for the treatment of the diabetic complications and related disses: these patents have been licensed and he has no financial interest in this work and declares no other duality of interest. All the other authors declare that they have no duality of interest in regard to this manuscript.

## Conflict of interest

None.

## Figures and Tables

**Table 1:** Metal concentrations of unmodified liver tissue from *ob/ob* mice and non-obese controls.

Metal	Reference isotope	Control (N = 7)	*ob/ob* (N = 10)	*P-*value*
Na (mmol/kg wet wt)	^23^Na	29.1(27.1-31.2)	22.0 (20.6-23.4)	<0.0001
Mg(mmol/kg wet wt	^24^Mg	11.3(9.9-12.7)	8.6(7.8-9.4)	0.0022
K(mmol/kg wet wt)	^39^K	97.4(90.4-104.3)	76.0(69.5-82.4)	0.0001
Ca(μmol/kg wet wt)	^44^Ca	754(672-835)	580(497-662)	0.003
Mn(μmol/kg wet wt)	^55^Mn	25.2(21.5-28.9)	13.2(11.8-14.6)	0.0001
Fe(μmol/kg wet wt)	^56^Fe	1055(891-1292)	730(624-836)	0.015
Cu(μmol/kg wet wt)	^63^Cu	106(93-119)	59(52.5-62.5)	<0.0001
Zn(μmol/kg wet wt)	^66^Zn	467(416-518)	379(337-421)	0.007
Se(μmol/kg wet wt)	^78^Se	20.8(17.9-23.8)	13.6(11.7-15.4)	0.0004
Mo(μmol/kg wet wt)	^95^Mo	10.8(9.5-12.2)	9.5(8.5-10.4)	0.067

Data are means (±95% CI). *Significance of between-group differences by Welch's modified *t*-tests.

**Table 2:** Metal concentrations in defatted-dried liver tissue from *ob/ob* mice and non-obese controls.

Metal	Reference isotope	control(N = 7)	*ob/ob*(N = 10)	*P-*value*
Na(mmol/kg defatted-dried wt)	^23^Na	28.3(18.8-37.8)	18.3(15.0-20.8)	0.047
Mg(mmol/kg defatted-dried wt)	^24^Mg	38.4(26.0-50.7)	35.4(32.6-38.3)	NS
K(mmol/kg defatted-dried wt)	^39^K	134(97-171)	104(91-117)	NS
Ca(mmol/kg defatted-dried wt)	^44^Ca	3.4(2.7-4.2)	3.0(2.8-3.3)	NS
Mn(μmol/kg defatted-dried wt)	^55^Mn	102(72-133)	68.9(63.9-74.0)	0.037
Fe(mmol/kg defatted-dried wt)	^56^Fe	4.3(3.3-5.4)	3.5(3.0-4.0)	NS
Cu(μmol/kg defatted-dried wt)	^63^Cu	502(406-597)	339(316-361)	0.0053
Zn(μmol/kg defatted dry wt)	^66^Zn	1.9(1.6-2.1)	1.9(1.8-2.1)	NS
Se(μmol/kg defatted dry wt)	^78^Se	75.9(63.6-88.5)	63.7(58.8-68.6)	0.059
Mo(μmol/kg defatted-dried wt)	^95^Mo	42.0(35.1-48.9)	47.6(45.0-50.3)	NS

Data are means (±95% CI). *Significance of between-group differences by Welch's modified *t*-test.
